# Preliminary efficacy investigations of oral fipronil against *Anopheles arabiensis* when administered to Zebu cattle (*Bos indicus*) under field conditions

**DOI:** 10.1016/j.actatropica.2017.07.030

**Published:** 2017-12

**Authors:** Richard M. Poché, Naftaly Githaka, Frans van Gool, Rebekah C. Kading, Daniel Hartman, Larisa Polyakova, Edward Okoth Abworo, Vishvanath Nene, Saul Lozano-Fuentes

**Affiliations:** a Genesis Laboratories, Inc., 10122 N.E. Frontage Road, Wellington, CO 80549, USA; b International Livestock Research Institute, P.O. Box 30709, Nairobi 00100, Kenya; c EXCELVET-Consultants, Twin Center, Tour Ouest, Angle Boulevard Zerktouni et El Massira 20000, Casablanca, Morocco

**Keywords:** Malaria, Vector, Systemic insecticide, One health, Kenya

## Abstract

Globally, malaria remains one of the most important vector-borne diseases despite the extensive use of vector control, including indoor residual spraying (IRS) and insecticide-treated nets (ITNs). These control methods target endophagic vectors, whereas some malaria vectors, such as *Anopheles arabiensis*, preferentially feed outdoors on cattle, making it a complicated vector to control using conventional strategies. Our study evaluated whether treating cattle with a capsule containing the active ingredient (AI) fipronil could reduce vector density and sporozoite rates, and alter blood feeding behavior, when applied in a small-scale field study. A pilot field study was carried out in the Samia District, Western Kenya, from May to July 2015. Four plots, each comprised of 50 huts used for sleeping, were randomly designated to serve as control or treatment. A week before cattle treatment, baseline mosquito collections were performed inside the houses using mechanical aspirators. Animals in the treatment (and buffer) were administered a single oral application of fipronil at ∼0.5 mg/kg of body weight. Indoor mosquito collections were performed once a week for four weeks following treatment. Female mosquitoes were first identified morphologically to species complex, followed by PCR-based methods to obtain species identity, sporozoite presence, and the host source of the blood meal. All three species of anophelines found in the study area (*An. gambiae s.s., An. arabiensis*, *An. funestus* s.s.) were actively transmitting *Plasmodium falciparum* during the study period. The indoor resting density of *An. arabiensis* was significantly reduced in treatment plot one at three weeks post-treatment (T1) (efficacy = 89%; T1 density = 0.08, 95% credibility intervals [0.05, 0.10]; control plot density = 0.78 [0.22, 0.29]) and at four weeks post-treatment (efficacy = 64%; T1 density = 0.16 [0.08, 0.14]; control plot density = 0.48 [0.17, 0.22]). The reduction of *An. arabiensis* mosquitoes captured in the treatment plot two was higher: zero females were collected after treatment. The indoor resting density of *An. gambiae* s.s. was not significantly different between the treatment (T1, T2) and their corresponding control plots (C1, C2). *An. funestus s.s.* showed an increase in density over time. The results of this preliminary study suggest that treating cattle orally with fipronil, to target exophagic and zoophagic malaria vectors, could be a valuable control strategy to supplement existing vector control interventions which target endophilic anthropophilic species.

## 1 Introduction

Malaria is the leading cause of morbidity in children in western Kenya (Maina et al., 2010). However, between 2000 and 2015, malaria incidences and mortality rates fell by 42% and 66% in Africa (Benelli and Mehlhorn, 2016, World Health Organization, 2015). Kenya’s nearly 8 million annual malaria cases are due to the most virulent malaria parasite, *Plasmodium falciparum* (Zhou et al., 2011). The three dominant malaria vectors in western Kenya, *An. gambiae s.s.* Giles*, An. arabiensis* Patton, and *An. funestus s.s.* Giles, have undergone changes in their abundance over the last ten years, most likely in response to the pressures of indoor residual spraying (IRS) and insecticide-treated nets (ITNs) (Bayoh et al., 2010, Lindblade et al., 2006). These strategies have led to the reduction of indoor biting (endophagic) and indoor resting (endophilic) vector populations, like *An. gambiae s.s.* and *An. funestus s.s*. In parts of western Kenya, these control measures have resulted in *An. arabiensis* becoming the most abundant malaria vector, while *An. gambiae s.s.* numbers have dramatically declined (Bayoh et al., 2010).

Even with ITNs increased coverage in western Kenya, a recent resurgence of malaria transmission suggests that this vector control method may no longer be effective at reducing malaria transmission (Zhou et al., 2011). When ITNs were still capable of reducing transmission in this region, the effect was only noticeable against *An. gambiae s.s.,* and *An. funestus s.s.* and not *An. arabiensis* (Mathenge et al., 2001). In many locations *An. arabiensis* preferentially feeds on cattle and rest outside of human habitations (Kweka et al., 2009, Mahande et al., 2007, Tirados et al., 2006), so they are unlikely to encounter interventions which target endophagic and endophilic mosquitoes. Furthermore, the resistance of *An. gambiae s.s. and An. arabiensis* mosquitoes in Western Kenya to pyrethroid compounds used for IRS and impregnated bed nets complicates their efficacy (Wanjala et al., 2015). Therefore, many reasons warrant development of novel vector control strategies that target residual malaria transmission driven by outdoor biting vectors. Targeting outdoor biting vectors is difficult, with the current options in urban areas being insecticidal fogs or ultra-low volume (ULV) sprays. However, these interventions are not practical in rural settings where much of the malaria transmission in Africa occurs, and they are prohibitively expensive for large scale application.

Preliminary data support the use of endectocides in cattle as an effective approach for the control of outdoor-biting vectors. A cattle-targeted insecticide strategy for control of the vectors of *Leishmania* spp. (*Phlebotomus argentipes and P. papatasi)* has already been explored in India and Tunisia (Derbali et al., 2014; Ingenloff et al., 2013; Poché et al., 2013). In western Kenya, *An. gambiae s.l.* were successfully controlled after feeding on cattle which had been injected with ivermectin (Fritz et al., 2009). Ivermectin and moxidectin administered by injection at doses of <600 μg/kg body weight (BW) reduced the survivorship and fecundity of *An. gambiae s.s*.; a 90% mortality was achieved for *An. gambiae s.s.* for up to 24 days post-treatment and complete elimination of egg production was observed for ten days following treatment (Fritz et al., 2012).

A different systemic drug, fipronil, works by blocking the GABA-gated ion channels in the central nervous system of arthropods (Raymond-Delpech et al., 2005). Fipronil is used to control ants, cockroaches, fleas, ticks, termites, and other insects. It is used in granular turf products, topical pet care products, gel baits, liquid termiticides, and agriculture (Roberts and Hutson, 1999, Tomlin, 2009). It also has been shown that fipronil can function as a systemic drug when applied to rodents and cattle. Fipronil is approved in many countries to control ectoparasites on domestic animals, and its safety to mammals and people is well established (Lee et al., 2010). Fipronil is approved for beef cattle in some countries as a pour-on application or in cattle dips, however, is not approved for use in dairy cattle. We took steps to prevent or eliminate the exposure of participants to fipronil.

In cattle, fipronil has successfully controlled leishmaniasis vectors for an extended period (Derbali et al., 2014; Ingenloff et al., 2013; Poché et al., 2013). In a laboratory setting, a single 0.5 mg/kg BW application has been shown to be effective against *An. arabiensis* for at least 7 days (Poché et al., 2015). However, field tests of fipronil for mosquito control are currently lacking. Therefore, the objective of this study was to evaluate the effect of treating cattle with fipronil on the resting density, blood-feeding behavior, and sporozoite rates of *Anopheles* vectors of malaria in western Kenya. Systemic circulation of fipronil, following treatment, has been shown to reduce mosquito survivorship after acquisition of a blood meal (Poché et al., 2015). We hypothesized that mosquito density would be significantly reduced after taking a blood meal from fipronil-treated cattle. Additionally, fipronil kills other blood-feeding ectoparasites including fleas (Poché et al., 2017) and ticks (Dolan et al., 2004) thereby improving animal health and increasing the sustainability of this “one health” approach.

## 2 Material and methods

### 2.1 Study area

The study took place in an area between Busia (34.11101° longitude, 0.45822° latitude) and Sisenye (33.987551° longitude, 0.157814° latitude) along the coast of Lake Victoria in southwestern Kenya. This region of Kenya is classified as a tropical wet-dry climate with average temperatures 19 29 °C, and average precipitation of 1200 mm per year (Pidwirny, 2011). A randomized block design was used for this experiment consisting of two sites (Site 1 and Site 2), each with one fipronil-treated plot and one untreated control plot (Fig. S1 of Supplementary data).

Site 1 and Site 2 were separated by at least 1 km. Within each site, treatment plots (T1 and T2) and control plots (C1 and C2) were separated by approximately 0.5 km. Additionally, plots were located at least 0.5 km from a large swamp and lake habitats. From each of the four plots, we enrolled 50 huts in which people were actively sleeping for a total of 200. Only houses with thatched roofs were selected to reduce the potential for internal temperature variation of tin roofs to affect the mosquito sampling. Buffer zones of 0.5 km surrounded the treatment plots and were calculated drawing the minimum convex polygon of the participating huts. The absence of cattle in the houses did not exclude huts from participation. All cattle from participating families located inside the treatment plots (T1, T2) as well as the surrounding buffer zones were treated.

Written informed consent was obtained from the head of the household for participation in this study. The participants signed a corresponding consent form for whether their homes were in a treatment area (mosquito collections and cattle treatments), a control area (mosquito collections only) or the buffer area (cattle treatments only). Consent forms were translated into KiSwahili and English. Homesteads or families, that declined involvement at the moment of consent or at any time during the study, were not included.

### 2.2 Cattle treatment

In treatment villages (T1-T2) all available healthy Zebu cattle (*Bos indicus*) (>∼6 months) were orally administered one capsule containing ∼0.5 mg/kg fipronil (CHEBI:5063, Gharda Chemicals Ltd., India). Treatment occurred one week following baseline mosquito collections. An experienced local veterinarian was present during the dosing to estimate the weight of the animals and remained on call in case of any adverse effects on the cattle. Efforts were also taken to prevent fipronil exposure to participants.

Even though evidence that accidental exposure to fipronil (in concentrations greater than in this study) presents only mild risks with temporary health effects (Lee et al., 2010), we advised the enrolled households that no treated animals be killed for meat within a week of treatment. We purchased all the milk produced by the participants’ cows and replaced it with locally available milk packets. This milk buy-and-replace program was carried out every day for 14 days after animal treatment.

### 2.3 Entomological impact

A collection of indoor resting mosquitoes was completed one week before cattle dosing to establish a baseline. Indoor mosquito collections were continued at one-week intervals for four weeks post-treatment. To determine the density of each mosquito species (Silver, 2008, Vazquez-Prokopec et al., 2009) collections were performed by aspiration catches (AC) from 6:30 am until 10:30 am Each collector spent five minutes per house using a mechanical aspirator (Prokopack), a chronometer, and a headlamp (1600 lumens) to collect mosquitoes. The collected mosquitoes were then transported from the field to a laboratory located at the International Livestock Research Institute (ILRI) in Busia for sorting and storing in silica-filled 0.6 ml tubes. Preserved mosquito samples were later shipped to Genesis Laboratories in Wellington, Colorado.

Lozano-Fuentes et al. (2016) attempted outdoor collection of mosquitos using clay pots but found the method to be inadequate. Consequently, we used indoor collections only.

### 2.4 Mosquito identification, blood meal source, and sporozoite rates

*Anopheles* mosquitoes were first sorted by sex and identified morphologically (Gillies and Coetzee, 1987, Gillies and De Meillon, 1968). Female mosquitoes morphologically identified as *An. gambiae* s.l. or *An. funestus* s.l. were later confirmed molecularly (Koekemoer et al., 2002, Scott et al., 1993).

DNA was extracted separately from mosquito abdomens and head/thoraces (Kent and Norris, 2005). Multiplex polymerase chain reaction (PCR) was performed on abdomens to detect the presence of single blood meals from humans, cattle, dogs, pigs, and goats (Kent and Norris, 2005). Head/thorax DNA extractions were screened for *P. falciparum* DNA using nested PCR (Snounou et al., 1993).

### 2.5 Data analysis of entomological impact parameters

#### 2.5.1 Bayesian parameter estimation

We selected Bayesian parameter estimation and credibility interval comparisons in place of Null Hypothesis Significance Testing (NHST) to avoid data transformation. The nature of the study data made it impossible to conform the required NHST assumptions of normality and equal variances across groups. The data also contained a large number of zeros placing our parameter estimates close to zero; therefore, using confidence intervals would result in nonsensical negative values. Bayesian inference is a robust method that takes advantage of the data structure without transformation and deals with unequal variances (Wasserstein and Lazar, 2016), is similar to Likelihood inference and can make use of previous knowledge, expressed mathematically, to adjust the likelihood function. The mosquito collection data are distributed as supplementary material should the reader prefer to use other estimation methodology.

#### 2.5.2 Blood meal proportions

It was assumed that blood meals (*y*) from each host (*k*) followed a multinomial distribution with an unknown proportion (*p*) of the total number of blood meals (*N*).

(1) y[1...k]∼Multinomial(p[1...k],N)

Bayesian inference can make use of prior knowledge to modify the likelihood function (1). The same weight was given to all possible values of *p* using a flat prior from a Dirichlet distribution, where the shape parameter α equal the inverse of the total number of blood meal hosts.

(2) $$p\left[1...k\right]∼Dirichlet\left(\alpha \left[1...k\right]=\frac{1}{k}\right).$$

The unknown parameter estimates (posterior distribution) were obtained using a Markov Chain Monte Carlo (MCMC) sampler with four chains, a burn-in of 10,000 (discarded iterations), 100,000 iterations, and a thinning of 250. We used the JAGS 4.0 library (Plummer, 2003) for the R 3.2 language (R Core Team, 2014). Convergence and autocorrelation were assessed using the CODA 0.18 R package (Plummer et al., 2006). The 95% credibility intervals (Kruschke, 2013) are equal to the 5th and 95th quantiles of the posterior distribution and represent the uncertainty of the calculated parameter; this range contains the parameter with a 95% probability. Overlapping credibility intervals show that the compared parameters are not statistically different given the uncertainty of the estimation.

#### 2.5.3 Sporozoite rate

We assumed *P. falciparum* positive/negative mosquitoes (*y*) followed a binomial distribution (*y* ∼ Binomial (θ, N)) with an unknown sporozoite rate (θ) from the total number of individuals tested (N). The same weight was given to all possible values of the sporozoite rate by selecting a non-informative prior from a Beta distribution (θ ∼ Beta (1, 1)). We used the same software and procedures as in the blood meal multinomial parameter estimation.

#### 2.5.4 Mosquito density

We estimated the mean number of indoor resting female *Anopheles* mosquitoes per hut at five-time points, (baseline, 1, 2, 3 and 4 weeks’ post-treatment) and four plots (T1, T2, C1, and C2). To achieve this, we assumed the number of female mosquitoes per hut (y) followed a Poisson distribution (*y* ∼ Poisson (λ)). The Poisson distribution is commonly used to describe randomly distributed objects (mosquitoes) counted through a randomly placed window (a hut). The Poisson distribution can be used to model only non-negative integers, and its shape is controlled by a single parameter (λ) that is equal to both the mean and the variance.

Non-informative priors were used for the baseline estimation (λ ∼ Log-normal (*μ* = 0, σ = 100). However, in the following weeks, the estimate from the previous week was used as priors (e.g. the posterior distributions of the baseline serve as prior for the one-week post-treatment estimation). The same software and procedures were employed as described for the blood meal parameter estimation. The estimated means were contrasted using 95% credibility intervals (Kruschke, 2013).

## 3 Results

### 3.1 Enrollment

Only one household withdrew from the study after originally giving their consent because the family moved out of the village, and was replaced with a nearby hut. From the enrollment surveys, it was estimated that the treatment plots had 277 sleepers (people actively sleeping in the buildings) while the control plots had 322 sleepers (Table 1). During enrollment, participants reported 151 cattle in the treatment, 134 in the control, and 1136 for both buffer zones (Table 1).

**Table 1:** Number of people and cattle per plot study site, and cattle body weight.

Sites	T1	C1	B1	T2	C2	B2
Sleepers	135	161	NA	142	161	NA
Cattle per plot	107	85	550	44	49	586
Mean Cattle BW (kg)	183 (163, 203)	NA	191 (184, 199)	115 (68, 153)	NA	168 (160, 176)

BW: Cattle Body Weight, (Body Weight ∼ Normal (μ, σ), μ ∼ Normal (0, 1000) and σ ∼ Log-Normal (0, 1000)). The values in parenthesis represent the 95% credibility intervals for the estimated cattle weight.

NA = Not Applicable, cattle weight was estimated during dosing.

### 3.2 Cattle treatment

A veterinarian estimated the weight of the animals before dosing with fipronil. Approximately 35% of the cattle weighed in the 151–250 kg range (36%, 95% credibility intervals [33%, 38%]). Cattle less than 251 kg comprised 78% of the treated cattle while heavier cattle were less common. In total, we treated 657 cattle in Site 1 (*i.e.* T1 and buffer zone), and 630 cattle in Site 2 (*i.e.* T2 and buffer zone). The cattle mean body weight was significantly higher in Site 1 than Site 2. Additionally, cattle within the T2 buffer zone were significantly heavier on average than cattle within inner T2.

### 3.3 Entomological impact

#### 3.3.1 Mosquito identification

After removing male mosquitoes and other arthropod taxa, 1558 mosquitoes were identified morphologically to  *An. gambiae s.l.* or *An. funestus s.l.* Molecular analysis confirmed 275 *An. arabiensis,* 392 *An. funestus s.s.*, and 613 *An. gambiae s.s.* The collection numbers by plot, species, and date are provided as supplemental data.

#### 3.3.2 Mosquito blood feeding patterns

Forty seven (47) of 423 tested samples contained blood meals that were identifiable. No *An. funestus* blood meals were PCR-positive despite using only engorged females.

**Fig. 1:**
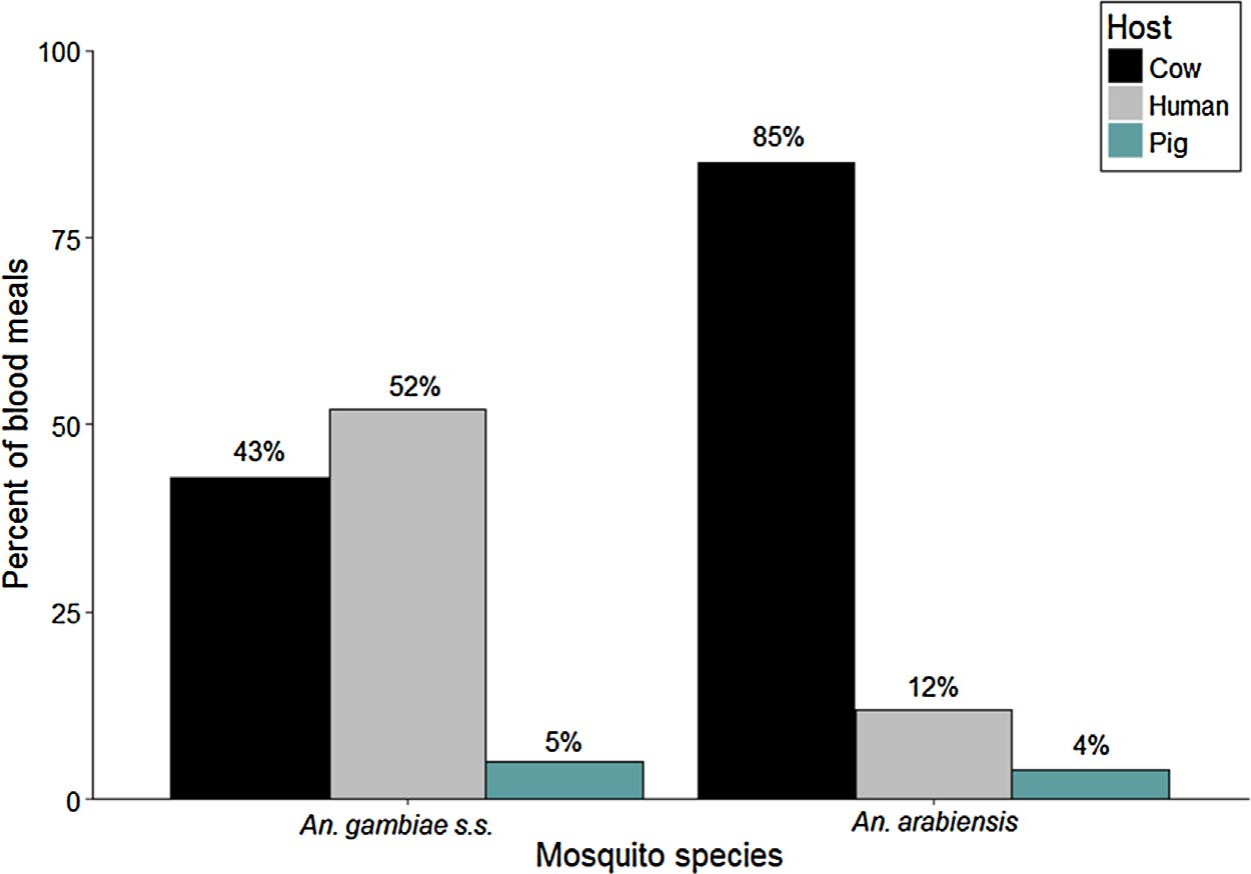
Mosquito blood feeding patterns.

We considered each blood meal a unique blood-feeding event. We found a total of 21 events for *An. gambiae s.s.* and 26 events for *An. arabiensis*. For *An. arabiensis*, 22/26 (∼85%) of blood meals came from cattle, 3/26 (∼12%) from humans, and 1/26 (∼4%) from a pig. (Fig. 1). In contrast for *An. gambiae s.s.*, 11/21 (52%) blood meals came from humans, 9/21 (43%) from cattle, and 1/21 (5%) from a pig (Fig. 1).

#### 3.3.3 Sporozoite rates

Overall, *An. gambiae s.s.* had a significantly higher *P. falciparum* sporozoite rate than *An. arabiensis* in the treatment (*An. gambiae s.s.* sporozoite rate = 0.10, 95% credibility intervals [0.07, 0.15]; *An. arabiensis* = 0.016 [4 × 10^−4^, 0.05]) and in the control plots (*An. gambiae s.s.* = 0.10 [0.07, 0.13], *An. arabiensis* = 0.022 [0.001, 0.05]). The highest rate was observed in *An. funestus s.s.* (treatment sporozoite rate = 0.12 [0.04, 0.21]; control sporozoite rate = 0.10 [0.03, 0.20]) but was not significantly different to the other species.

##### 3.3.3.1 *Anopheles arabiensis* sporozoite rate

A total of three (of 68) *An. arabiensis* (4.4%) within T1 during weeks one, three, and four post-treatment, all collected from different huts, were *P. falciparum* sporozoite-positive. No sporozoite-positive *An. arabiensis* mosquitoes were obtained from either control plot (C1, C2). We were unable to estimate a sporozoite rate for T2 since no female *An. arabiensis* mosquitoes were collected after treatment and the single female collected during the baseline was negative.

##### 3.3.3.2 *Anopheles funestus s.s.* sporozoite rate

*An. funestus s.s.* females were collected during the final 2–3 weeks of the study only. The overall rate was 0.10 [0.04, 0.16] (9 positive/101 tested). In Site 1 positive samples were only collected during the final week of the study (T1, 2/11 ∼ 0.22 [0.036, 0.45]; C1, 4/9 ∼ 0.45 [0.17, 0.72]). In Site 2 only the three weeks post-treatment collections had positive samples (T2, 2/7 ∼ 0.32 [0.058, 0.61]; C1, 1/19 ∼ 0.08 [0.003, 0.22]).

##### 3.3.3.3 *Anopheles gambiae s.s.* sporozoite rate

**Fig. 2:**
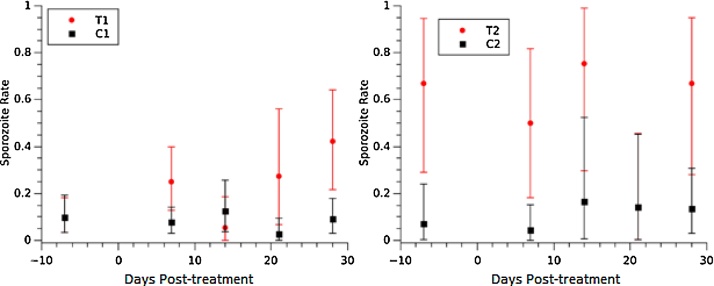
*An. gambie* s.s. sporozoite rate. Dosing of cattle was conducted at Day 0. The bars represent 95% credibility intervals around the sporozoite rate.

We consistently collected *P. falciparum* sporozoite-positive *An. gambiae* s.s in three of the study plots (T1, C1, and T2). C2 was active in only one of the five weekly collections while the other plots were active in four out of five (Fig. 2). Both study sites showed significant differences in *An. gambiae s.s.* sporozoite rates between the treatment and corresponding control plots in at least one of the weekly collections. In particular, these statistical differences were observed during the last week in Site 1, and during the first and second weeks post-treatment in Site 2 (Fig. 2).

The small samples size caused considerable uncertainty in the sporozoite rate estimations. However, in C1 we continuously collected a sizeable number of female *An. gambiae* s.s increasing the precision. The estimated upper and lower limits from C1 suggest that the sporozoite rate ranged between 2% and 25% in 1, 2 and 4 weeks post-treatmentwhile the sporozoite rate ranged between <1% and 9% in week-3 post-treatment (Fig. 2). These results show that detecting changes in mosquito sporozoite rates in small time scales require larger sampling efforts, dramatic reductions (or increases) in positive females, or refinements of the estimation model.

#### 3.3.4 Mosquito resting density

**Fig. 3:**
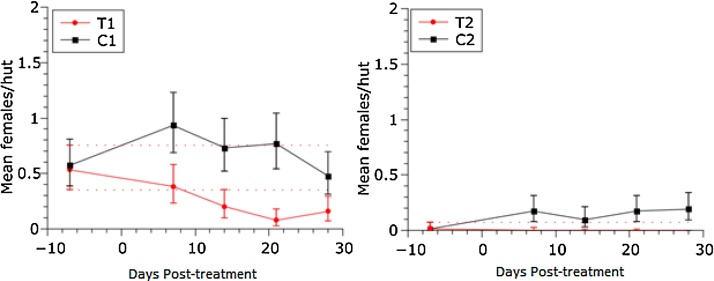
Effect of fipronil on *An. arabiensis* indoor resting density. The vertical bars represent 95% credibility intervals around the mean. Dotted red lines represent the upper and lower limits of the credibility intervals for the baseline. (For interpretation of the references to colour in this figure legend, the reader is referred to the web version of this article.)

**Fig. 4:**
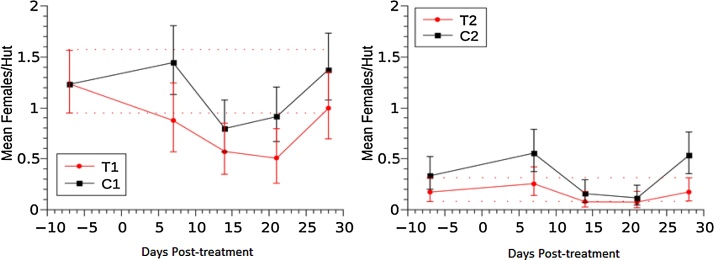
Effect of fipronil on *An. gambiae* s.s. indoor resting density.

**Fig. 5:**
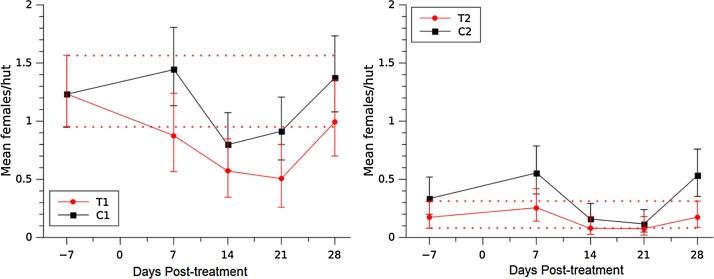
Effect of fipronil on *An. funestus* s.s. indoor resting density.

Overall, *An. gambiae s.s.* presented the highest density of all species, with its highest density in Site 1. During the baseline, *An. arabiensis* and *An. gambiae s.s.* did not show significant differences in density when comparing treatment and control plots within each study site (Fig. 3, Fig. 4). However, the indoor resting density of *An. funestus s.s* was significantly different in Site 1 but not Site 2 (Fig. 5).

##### 3.3.4.1 Anopheles arabiensis density

One week after dosing, significant differences in the resting densities of *An. arabiensis* were observed between the control and the treatment plots within each study site. *An. arabiensis* was only captured on T2 during the baseline (a single female); after treatment, none of the sampled huts produced any *An. arabiensis* (Fig. 3). Within Site 1, T1 was significantly different to its corresponding baseline (i.e. the 95% credibility intervals for T1 did not overlap with the dotted red line; Fig. 3). The density on T1 was near to significantly different at 2 weeks post-treatment, and at 3 weeks post-treatment the difference became significant (95% probability) and remained significant for at least 4 weeks post-treatment (Fig. 3). The observed density reductions, using the Henderson & Tilton formula (Henderson and Tilton, 1955), represent efficacies in the treatment of 89% and 64% at weeks-3 and 4 post-treatment, respectively. In C2 the density remained the same after dosing while in T2 we did not find a single female after the treatment.

##### 3.3.4.2 *Anopheles funestus s.s.* density

*An. funestus s.s.* also showed variation in the mean number of females per hut after treatment. On Site 1 (Fig. 5) indoor resting density increased significantly between the baseline and the following weeks, whereas in Site 2, indoor resting density was statistically similar in T2 and C2.

##### 3.3.4.3 *Anopheles gambiae s.s*. density

After the dosing, there were changes in the mean density of *An. gambiae s.s.* within both study sites. The density was higher at one and four weeks post-treatment but lower during weeks two and three post-treatment (Fig. 4) from the baseline. On T1, these changes were significantly different from the baseline at weeks three and four. However, the reductions were not significantly different to their corresponding control plot. A similar pattern was observed on Site 2 but the density reductions for at weeks 3 and 4 post-treatment were not significantly different from baseline values.

## 4 Discussion

This study evaluated the use for fipronil, orally administered to village cattle, against *An. arabiensis* under field conditions in western Kenya. The efficacy of fipronil against *An. gambiae s.l.* has been tested previously in the laboratory and cattle shed experiments (Burruss et al., 2014; Poché et al., 2015). To the best of our knowledge, this study is the first to assess the efficacy of fipronil presented orally under field conditions for malaria vector control. Fipronil has long been used for controlling ectoparasites in pets (Jackson et al., 2009), and its effectiveness as a public health pesticide should be fully investigated. Residues of fipronil in cattle blood were reported to remain for up to 35 days (below international allowable limits) (Poché et al., 2013).

The area of western Kenya where this study was conducted experiences active hyperendemic transmission of *P. falciparum* driven by *An. gambiae s.s.*, *An. arabiensis*, and *An. funestus* s.s (CDC, 2015, Division of Malaria Control, 2011, Lozano-Fuentes et al., 2016, Zhou et al., 2011). In the current study, the indoor resting density of these mosquito species was consistently higher on Site 1. It is possible that this is the result of Site 1 being closer to the delta of the Nzoia River which may provide a larger number of breeding sites in comparison to the lakeshore habitats near Site 2. Mwangangi et al. (2007) found that swamp habitats, like the ones found along the coast of Lake Victoria, are less productive mosquito breeding habitats than areas with streams, pools and puddles present along the shores of rivers and deltas.

During this study, the treatment of cattle with fipronil had a delayed effect on the indoor resting density of *An. arabiensis*. Given the complex population dynamics, we used two *ad hoc* criteria to determine if the changes in population density within the treatment plots (T1, T2) were significant: a statistically significant difference within the corresponding control plot, and at the same time, a significant difference with the baseline values. Using this conservative approach, we can conclude that there was a reduction of 89% in the resting density of *An. arabiensis* at week-3 post-treatment and 64% at week-4 post-treatment. Using a less stringent criterion, a significant difference only to the control plot, we could consider the first and the second weeks post-treatment to have significant reductions of 55% and 70% respectively. This density reduction was expected since *An. arabiensis* feeds 81–89% on cattle in the study area (Lozano-Fuentes et al., 2016).

*Anopheles gambiae s.s.* did not show the same significant reduction in resting density using our stringent criteria, suggesting that this species is less affected by this mosquito control strategy. Given the higher degree of anthropophily of *An. gambiae s.s.* compared with *An. arabiensis* in this study area (Lozano-Fuentes et al., 2016), this result is not surprising. However, the T1 plot showed a significant difference in the indoor resting density of *An. gambiae s.s.* during weeks three and four post-treatment when compared with the baseline. We hypothesize that these reductions in T1 may be due in part to fipronil-treatment because *An. gambiae s.s.* fed on cattle between 14 and 27% (Lozano-Fuentes et al., 2016) of the time in our study area. This density reduction could also be the result of changing the local mosquito population dynamics. Paaijmans et al. (2009) found that the larval development time of *An. gambiae s.s.* decreased when *An. arabiensis* was also present at the breeding site in Western Kenya (Paaijmans et al., 2009). Therefore, any reduction in *An. arabiensis* as a result of treating cattle with fipronil may have had an indirect effect on the density of *An. gambiae s.s.* through the dynamics of larval competition.

We did not anticipate any effect of cattle dosing on *An. funestus s.s.*, since the species only feeds on humans in this area (Lozano-Fuentes et al., 2016). Accordingly, the indoor resting density of this species appears not to have been effected by the fipronil dosing of cattle, as indoor resting densities increased significantly in plots T1, C1, and C2. This pattern is consistent with the habitat selection of *An. funestus s.s.* since it prefers small puddles which were not present in the baseline collections, but increased throughout the study period as the rainy season ended and more breeding sites became available (Mwangangi et al., 2007).

The results of this study revealed that investigations are necessary to explore the interspecies dynamics that may come into play when one species is virtually eliminated from a habitat. Bayoh et al. (2010) documented a decrease in the population of *An. gambiae s.s.* over multiple years in western Kenya due to the use of ITNs and a resulting proportional increase of *An. arabiensis*. In the current study, a decrease in the resting density of *An. arabiensis* was observed concurrently with an increase in the resting density of *An. funestus s.s.* (Fig. 3, Fig. 5). However, it is unclear whether or not these observations are related. Previously, Lozano et al. (2016) also reported an increase in the resting density of *An. gambiae s.s.* when the population of *An. arabiensis* was temporarily reduced following the treatment of cattle with eprinomectin. If there is a causal relationship between the treatment applied and the observed changes in the relative abundance of non-targeted malaria vectors, an integrated approach will be necessary (such as treating cattle in combination with ITNs, IRS, or human prophylactics) to counteract any increase in exposure by other anophelines. Therefore, monitoring the long-term fluctuations in relative abundance among these species, as a result of various malaria control interventions, is paramount.

The observed range of fipronil effectiveness has been obtained previously against fleas for up to 90 days post-treatment (Bonneau et al., 2010, McCall et al., 2004, Medleau et al., 2003). Similarly, ivermectin, when given to people, showed a 33.9% reduction in survivorship of *An. gambiae s.s.* for seven days following mass drug administration (Alout et al., 2014). While the effect was brief in the case of ivermectin, a significant reduction in mosquito parity rates was observed for more than two weeks following treatment, and sporozoite rates were reduced by 77.5% for 15 days (Alout et al., 2014). In this study, we did not follow entomological parameters other than mosquito density, host preference, and sporozoite rate. Thus measuring mosquito parity and longevity parameters in future field studies is necessary.

## 5 Conclusions

When cattle were treated with the drug fipronil, the indoor resting density of *An. arabiensis* was decreased by 89% at 3 weeks post-treatment. This reduction as a result of fipronil dosing was observed not only when compared to the baseline collections but also in comparison to mosquito densities in the control plots. This is noteworthy because malaria epidemics in Western Kenya, and many African regions, are driven in part by *An. arabiensis* that feed primarily on cattle.

Follow-up studies should assess the effect of a sustained cattle treatment regimen, evaluating the long-term impact on entomological and epidemiological indicators in combination with other control strategies. Such studies should also examine the inter-species dynamics between the anophelines in response to vector-control interventions that target one and/or the other species.

Village-wide treatment of cattle with fipronil has the potential to impact residual outdoor malaria transmission driven by *An. arabiensis* and may become a valuable tool to supplement existing vector control interventions which target the more anthropophilic species.

## Conflict of interests

The authors declare that they have no competing interests.

## Funding

This project was funded by the Bill and Melinda Gates Foundation, Global Health GrantNumber OPP1032369.

## Ethics approval and consent to participate

This study was approved by ILRI’s Animal Care and Use Committee with reference number IACUC2015.09. and ILRI’s Institutional Research Ethics Committee with reference number IREC2015.05.

## Availability of data and material

Mosquito collections by species, hut, and date are available at https://figshare.com/s/2b845e7d5155519658ce. Other data will be made available upon request.

## Author contributions

The project was conceived and designed by RP. Principal Investigators were SLF (Genesis), and NG (ILRI). The Study Director was SLF. The field work was conducted by SLF (Genesis), NG (ILRI), LP, and FVG (EXCELVET). LP verified the purity and concentration of the active ingredient. The data were analyzed and figures generated by SLF. The manuscript was written by SLF, RCK, and RP.
